# Prevalence of Non-Polio Enteroviruses in the Sewage of Guangzhou City, China, from 2013 to 2021

**DOI:** 10.1128/spectrum.03632-22

**Published:** 2023-03-30

**Authors:** Shufen Huang, Yong Zhang, Wei Zhang, Meizhong Chen, Caixia Li, Xue Guo, Shuangli Zhu, Hanri Zeng, Ling Fang, Bixia Ke, Hui Li, Hiromu Yoshida, Wenbo Xu, Xiaoling Deng, Huanying Zheng

**Affiliations:** a Guangdong Provincial Center for Disease Control and Prevention, Guangdong Workstation for Emerging Infectious Disease Control and Prevention, Panyu District, Guangzhou, China; b School of Public Health, Southern Medical University, Baiyun District, Guangzhou, China; c WHO WPRO Regional Polio Reference Laboratory and Ministry of Health Key Laboratory for Medical Virology, National Institute for Viral Disease Control and Prevention, Chinese Center for Disease Control and Prevention, Changping District, Beijing, China; d Department of Virology II, National Institute of Infectious Diseases, Tokyo, Japan; Xinxiang Medical University

**Keywords:** epidemiological trends, non-polio enteroviruses, sewage surveillance

## Abstract

Continuous surveillance of enteroviruses (EVs) in urban domestic sewage can timely reflect the circulation of EVs in the environment and crowds, and play a predictive and early warning role in EV-related diseases. To better understand the long-term epidemiological trends of circulating EVs and EV-related diseases, we conducted a 9-year (2013 to 2021) surveillance study of non-polio EVs (NPEVs) in urban sewage in Guangzhou city, China. After concentrating and isolating the viruses from the sewage samples, NPEVs were detected and molecular typing was performed. Twenty-one different NPEV serotypes were identified. The most isolated EVs were echovirus 11 (E11), followed by coxsackievirus (CV) B5, E6, and CVB3. EV species B prevailed in sewage samples, but variations in the annual frequency of different serotypes were also observed in different seasons, due to spatial and temporal factors. E11 and E6 were detected continuously before 2017, and the number of isolates was relatively stable during the surveillance period. However, after their explosive growth in 2018 and 2019, their numbers suddenly decreased significantly. CVB3 and CVB5 had alternating trends; CVB5 was most frequently detected in 2013 to 2014 and 2017 to 2018, while CVB3 was most frequently detected in 2015 to 2016 and 2020 to 2021. Phylogenetic analysis showed that at least two different transmission chains of CVB3 and CVB5 were prevalent in Guangzhou City. Our results show that in the absence of a comprehensive and systematic EV-related disease surveillance system in China, environmental surveillance is a powerful and effective tool to strengthen and further investigate the invisible transmission of EVs in the population.

**IMPORTANCE** This study surveilled urban sewage samples from north China for 9 years to monitor enteroviruses. Samples were collected, processed, and viral identification and molecular typing were performed. We detected 21 different non-polio enteroviruses (NPEVs) with yearly variations in prevalence and peak seasons. In addition, this study is very important for understanding the epidemiology of EVs during the COVID-19 pandemic, as the detection frequency and serotypes of EVs in sewage changed considerably around 2020. We believe that our study makes a significant contribution to the literature because our results strongly suggest that environmental surveillance is an exceptionally important tool, which can be employed to detect and monitor organisms of public health concern, which would otherwise be missed and under-reported by case-based surveillance systems alone.

## INTRODUCTION

Enteroviruses (EVs) are members of the genus *Enterovirus* and belong to the *Picornaviridae* family; they are small viruses with single positive-strand RNA genomes with icosahedral capsids (1, 2). A total of 116 EV types infecting humans are categorized into four species, named *Enterovirus A* (EV-A), *Enterovirus B* (EV-B), *Enterovirus C* (EV-C), and *Enterovirus D* (EV-D) (3). EVs are common human pathogens that infect millions of people worldwide every year. They are mainly transmitted through the fecal oral route in infants and children leading to a range of clinical manifestations. Although most infections are asymptomatic or result in only mild symptoms, sometimes they can also cause various other severe, potentially fatal diseases, such as aseptic meningitis, myocarditis, acute flaccid paralysis (AFP), and neonatal sepsis-like disease, complicating the clinical diagnosis of EV infections (4–6). Therefore, comprehensive surveillance and precise investigation of the prevalence and spread of EVs are very important for the control and intervention of EV-related diseases.

To prevent and control the outbreak of EV-related diseases, countries globally have established various EV surveillance networks. Laboratory-based surveillance is a strategy for the prevention, control, elimination, and eradication of infectious diseases (7). Surveillance is based on rapid case detection, reporting, and epidemiological and laboratory investigations. For instance, following the adoption of a global goal to eradicate polio by the World Health Assembly in 1988, the World Health Organization developed the Global Polio Laboratory Network (GPLN) to ensure high-quality laboratory diagnosis of suspected cases of poliomyelitis (8). GPLN is fully integrated with a case-based acute flaccid paralysis (AFP) surveillance system to provide poliovirus case confirmation; intratype identification between vaccine-derived polioviruses, vaccine-related polioviruses, and wild-type polioviruses; and molecular epidemiological data to guide immunization activities (9, 10). In 2017, an Asia Pacific EV surveillance network was established to estimate the disease burden, understand virus evolution, and promote vaccine development by coordinating laboratory diagnosis and data collection (11), and the European Non-Polio Enterovirus Network has been recently established to develop and share diagnostic technical knowledge on EV detection and characteristics, disease manifestations and prognosis, virus evolution, and pathogenesis (12). These EV surveillance networks will enable comparison of EV infection data across Asia and Europe, respectively. These data will also be used to determine the burden of EV infection, which is necessary to guide the prevention measures and policies of EV-related diseases (13).

Although some EV surveillance networks have been established worldwide, most are based on case surveillance systems. For example, the main enterovirus surveillance system in China includes AFP case surveillance system and hand, foot, and mouth disease case surveillance system. Importantly, most EV infections are non-symptomatic; therefore, it is difficult to track all EV infections through case surveillance systems. Owing to this, EV environmental surveillance was developed because it can find the “hidden” circulating EV serotypes in the community: this can be used as an important supplement to the EV disease surveillance system (14–17).

In this study, we provided an overview of the circulation patterns of the predominant non-polio EVs (NPEVs) in urban sewage in Guangzhou, China, from 2013 to 2021. Moreover, we combined the results of previous studies (2009 to 2012) for a comprehensive analysis (15) to better understand the long-term changes in EV disease trends and the epidemiological characteristics of circulating EVs during the 13 years.

## RESULTS

### NPEV serotypes.

**FIG 1:**
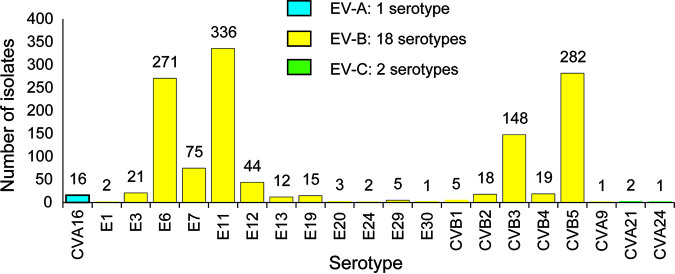
NPEV serotypes detected in the sewage of Guangzhou City, China, from 2013 to 2021.

Twenty-one NPEV serotypes were identified based on molecular typing of the 350-bp fragment sequence of the *VP1* region (Fig. 1). Most serotypes belonged to EV-B (1,260/1,279 [98.52%], including 18 EV serotypes), followed by EV-A (16 [1.25%], with only one serotype), and EV-C (three [0.23%], with two serotypes). Echovirus (E)11 was the most common serotype accounting for 336 (26.27%) of 1279 EVs, followed by coxsackievirus (CV)B5 (282; 22.05%), E6 (271; 21.19%), CVB3 (148; 11.57%), E7 (75; 5.86%), and E12 (44; 3.44%) (Table 1). These six most prevalent EV serotypes represented 90.38% of all typed EV-positive samples. Due to extremely large hygiene control at the subnational level during the COVID-19 pandemic, the variety of serotypes has been reduced up to only three, compared with 16 in 2013 (16, 12, 11, 12, 8, 8, 8 from 2013 to 2019, respectively) (Table 1). Then due to mitigation for COVID-19, numbers increased from three in 2020 to five in 2021. Also, the frequency of detection for EVs is still low level in 2020 and 2021.

**TABLE 1:** NPEV serotypes detected in the sewage of Guangzhou City (China), each year from 2013 to 2021^*a*^

Serotype	No. of EVs detected									
	2013	2014	2015	2016	2017	2018	2019	2020	2021	Total (%)
EV-A										16 (1.25)
CVA16	0	16	0	0	0	0	0	0	0	16 (1.25)
EV-B										1,260 (98.51)
E11	37	24	22	17	26	75	134	0	1	336 (26.27)
CVB5	30	130	2	2	30	86	1	0	1	282 (22.05)
E6	59	52	49	16	24	43	25	1	2	271 (21.19)
CVB3	4	1	22	31	1	0	0	22	67	148 (11.57)
E7	35	8	2	0	9	3	18	0	0	75 (5.86)
E12	22	7	9	5	0	0	1	0	0	44 (3.44)
E3	5	1	4	8	0	0	0	1	2	21 (1.64)
CVB4	1	0	0	10	1	5	2	0	0	19 (1.48)
CVB2	1	0	2	6	6	1	2	0	0	18 (1.41)
E19	8	3	0	0	2	1	1	0	0	15 (1.17)
E13	1	2	7	2	0	0	0	0	0	12 (0.94)
E29	1	4	0	0	0	0	0	0	0	5 (0.39)
CVB1	0	0	0	2	0	3	0	0	0	5 (0.39)
E20	1	1	0	1	0	0	0	0	0	3 (0.23)
E24	2	0	0	0	0	0	0	0	0	2 (0.16)
E1	0	0	2	0	0	0	0	0	0	2 (0.16)
E30	0	0	1	0	0	0	0	0	0	1 (0.08)
CVA9	1	0	0	0	0	0	0	0	0	1 (0.08)
EV-C										3 (0.24)
CVA21	2	0	0	0	0	0	0	0	0	2 (0.16)
CVA24	0	0	0	1	0	0	0	0	0	1 (0.08)
Total	210	249	122	101	99	217	184	24	73	1,279
(No. serotype)	16	12	11	12	8	8	8	3	5	

a EVs, enteroviruses; NPEV, non-polio enterovirus.

### Seasonal distribution characteristics.

**FIG 2:**
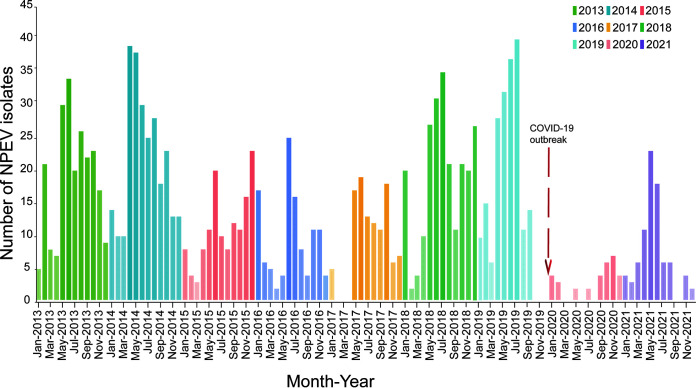
Number of NPEV isolates detected each month by virus isolation from 2013 to 2021. Different colors represent different years, and the shade of color represents the number of isolates.

Most EV-positive samples were obtained in summer months (Fig. 2). Overall, the number of EV-positive samples that were typed typically increased from May and peaked in June and July; however, in 2014, 2015, 2020, and 2021, the peak months were April (*n* = 38), December (*n* = 22), November (*n* = 6), and May (*n* = 2), respectively. Some variations in the seasonal prevalence of NPEVs were observed. As indicated in Fig. 2, the peaks in the number of isolates in 2014 and 2021 were brought forward to spring, while 2015, 2016, 2017, and 2018 had two peaks in summer and winter. Additionally, the number of NPEVs also increased in winter in 2020 and peaked in December. In 2018 and 2019, numerous NPEVs were isolated from May to July (90 and 106 isolates in total), and numerous (65 isolates in total) NPEVs were isolated from October to December 2018. However, a few NPEVs were detected in 2020 (only 24 isolates), possibly due to the prevalence of COVID-19. Our results differ from those of other studies (18, 19), in which the number of NPEVs in Guangzhou sewage samples did not all remain high during the summer and autumn.

### Epidemic characteristics of predominant EV serotypes.

**FIG 3:**
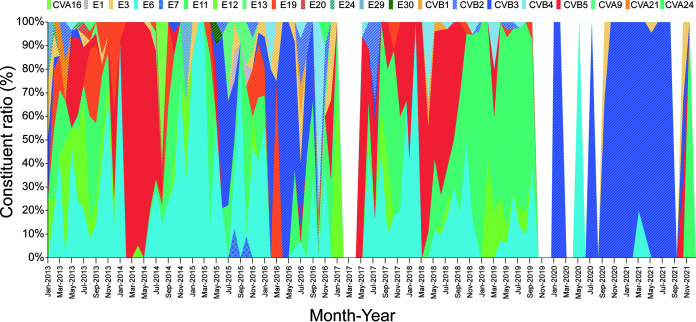
The percentage of each serotype isolated per month from 2013 to 2021. Different colors represent different serotypes.

**FIG 4:**
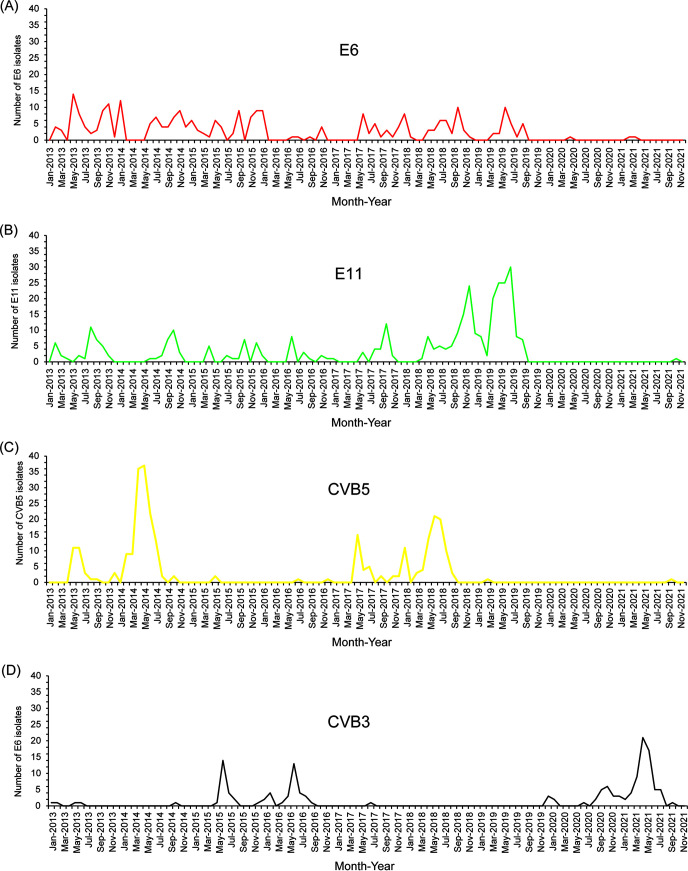
Seasonal patterns of four predominant circulating NPEVs in Guangzhou from 2013 to 2021. The serotypes are shown in Figures (A), (B), (C), and (D) are E6, E11, CVB5, and CVB3, respectively.

Notably, the distribution pattern of NPEV serotypes and the predominant serotypes also changed over the years. E6, E11, CVB5, and CVB3 were the most common serotypes during the study period (Table 1; Fig. 3), and E6 was isolated annually for 13 years. It reached the peak of strain isolation in 2010 (101 isolates) and was the most common serotype in 2010, 2013, and 2015 (Fig. 3), and then gradually declined since 2019 (Fig. 4A). E11 was generally detected before 2017, and the number of isolates was relatively stable, with a peak in summer and autumn. However, after its explosive growth in 2018 and 2019 (accounting for 34.56% and 72.83% of the total number of annual isolates, respectively), E11 abruptly disappeared. It was not detected in 2020, and only one E11 strain was detected in 2021 (Fig. 4B). CVB5 was rarely detected in 2011 and 2012, but the number of CVB5 detected increased suddenly in 2014 and 2018 (accounting for 52.21% and 39.63% of the total number of isolates in the whole year, respectively) and then decreased again (Fig. 4C). In contrast, no CVB3 isolates were detected in 2018 and 2019, but the number of CVB3 isolates detected increased significantly from 2020 (*n* = 22) to 2021 (*n* = 67), accounting for 91.67% and 91.78% of the total number of isolates detected for the respective years (Fig. 4D).

### *VP1* sequence analysis of CVB5.

**FIG 5:**
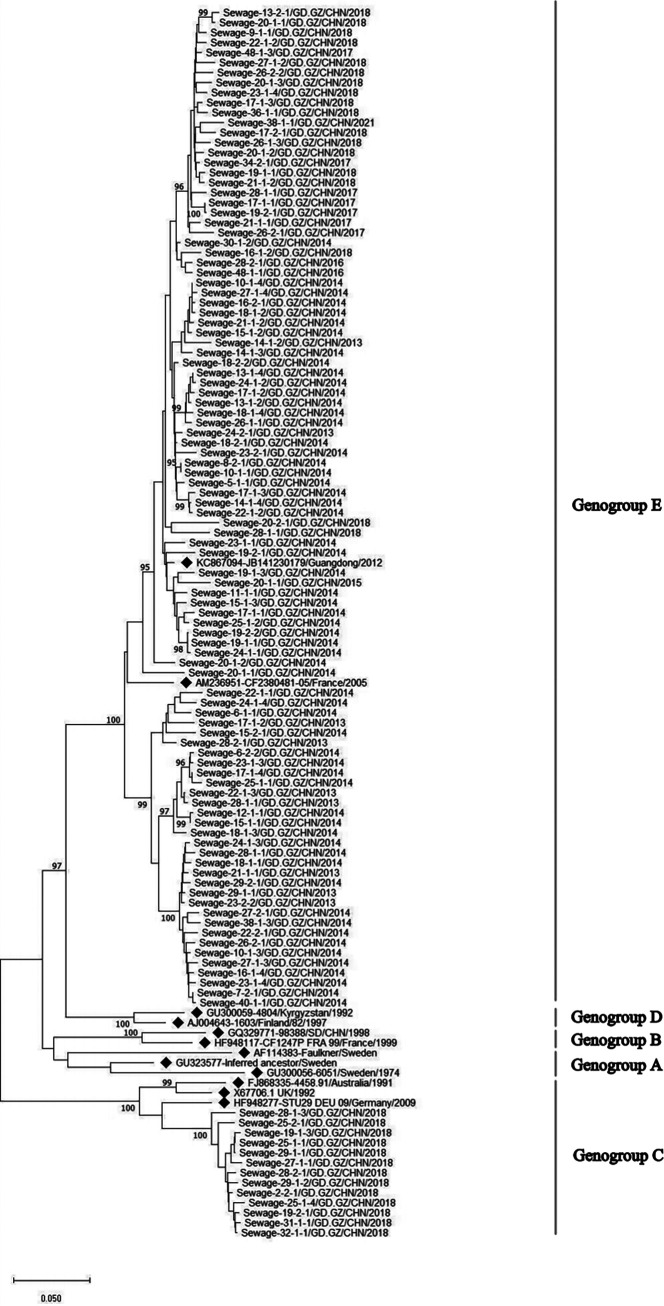
Phylogenetic tree based on VP1 sequence of Coxsackievirus B5 (CVB5) isolated in the sewage of Guangzhou City from 2013 to 2021. Symbol of solid diamond represents the reference sequence.

In 2013 to 2021, a total of 282 CVB5 strains were isolated in Guangzhou sewage surveillance, and entire *VP1* region nucleotide sequences of 849 nt were obtained, encoding 283 ammo acids. According to the preliminary sequence analysis and phylogenetic analysis, 111 representative sequences (GenBank number: OQ352015-OQ352125) from 2013 to 2021 were selected for analysis. The identity comparison showed that the sewage CVB5 sequences had 76.2% to 100% nucleotide identity and 94.6% to 100% amino acid identity, respectively. The nucleotide identity and amino acid identity of CVB5 and prototype strain Faulkner were 77.1% to 83.8% and 95.0% to 96.8%, respectively. In addition to some genotype C strains in 2018, the sewage CVB5 strains from 2013 to 2021 belonged to genotype E (Fig. 5).

### *VP1* sequence analysis of CVB3.

**FIG 6:**
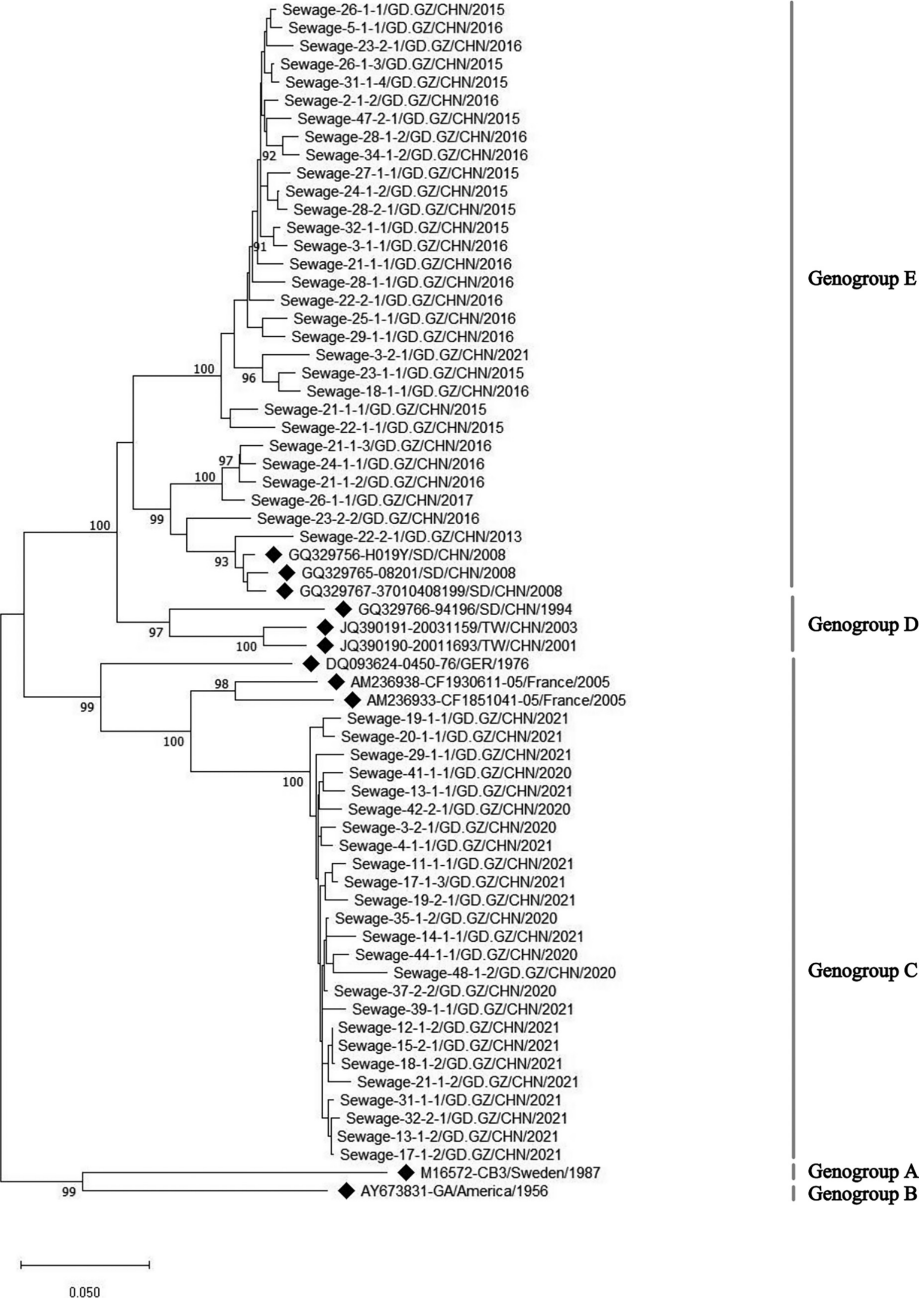
Phylogenetic tree based on VP1 sequence of Coxsackievirus B3 (CVB3) isolated in the sewage of Guangzhou City from 2013 to 2021. Symbol of solid diamond represents the reference sequence.

In 2013 to 2021, a total of 148 CVB3 strains were isolated in Guangzhou sewage surveillance. The identity comparison showed that the nucleotide sequence identity of *VP1* between CVB3 isolates from sewage surveillance was 78.9% to 100%, and the identity with the prototype strain of CB3 was 76.4% to 80.1%. Phylogenetic analysis was performed based on the 852-nt *VP1* sequences, and 55 representative sequences (GenBank number: OQ351960-OQ352014) from 2013 to 2021 were selected for analysis. CVB3 isolated before 2019 belonged to genotype E, but it belongs to genotype C since 2020 (Fig. 6). Therefore, the phylogenetic tree indicates that a new CVB3 transmission chain may emerge in Guangzhou in 2020, leading to the prevalence of new genotypes.

## DISCUSSION

To the best of our knowledge, this is the longest systematic study of EV prevalence in sewage in China, which reports the predominant NPEV serotypes circulating in environmental samples, emphasizing the important role of environmental surveillance in tracking EVs circulating in the population. Although a relatively uniform poliovirus surveillance program has been established worldwide to inform and evaluate vaccination and elimination programs, NPEV surveillance differs substantially between countries: for example, surveillance in the United States is based on voluntary reporting of EV typing data (20, 21) whereas in Europe, NPEV surveillance typically focuses on EVs detected in severely infected patients hospitalized with neurological symptoms (22). Notably, Japan has adopted a sentinel-based surveillance system for NPEVs that captures patients with aseptic meningitis; hand, foot, and mouth disease; herpangina; and acute hemorrhagic conjunctivitis (23). In contrast, EV surveillance in China is mainly based on hand, foot, and mouth disease and AFP case surveillance, because these two are notifiable infectious diseases in China (24–26). However, most human EV infections are generally asymptomatic, making it difficult to trace the infections through disease surveillance. Therefore, environmental surveillance is a sensitive method to detect silently circulating viruses, which should be regarded as a supplementary assessment tool to track the prevalence of EVs in the population.

In this study, we reported an overview of NPEV prevalence in the sewage of Guangzhou city, China. During the 9-year surveillance study (2013 to 2021), four common circulating NPEV serotypes were identified: E11, CVB5, E6, and CVB3 (in descending order). In contrast, E20, E24, E1, CVA21, E30, CVA9, and CVA24 were only occasionally detected in sewage samples; these results were similar to those of previous studies (15). Our results are similar to those of other studies: members of EV-B were identified most frequently, followed by EV-A and EV-C. Members of EV-D are rarely reported in municipal wastewater (18, 27, 28). Because we used the supernatants obtained after centrifugation of sewage for virus isolation, viruses combined with sediment in sewage could not be detected. Moreover, some viruses may exist in sewage below the detection limit, so some EV transmission may not be detected. Moreover, in our previous study, the cell lines used were proven to be conducive to the isolation of EV-B (15). Due to the limited range of EV species observations in sewage surveillance study and the dominance of EV-B detections, the overall EV species distribution has not been well-described on a seasonal basis, but EV-B is still generally found to be the most prevalent in summer and autumn.

Although the number of EV serotypes isolated is limited, the advantage of environmental surveillance is that it can use sewage samples to monitor large populations and can dynamically track the ecological changes of EV diversity in a region by monitoring these changes over time (18). In this study, E11, CVB5, E6, and CVB3 were the four most commonly detected serotypes. E11 is one of the most common causes of meningitis in the world; cases of E11 related to meningitis; AFP; and hand, foot and mouth disease have been detected in Shandong, Fujian, Yunnan, and other provinces in China (29, 30). In this study, E11 was most frequently isolated from 2018 to 2019. From 2009 to 2017, the dominant transmission chain of E11 was A1 genotype, but there were also C3 and D5 genotypes. Since 2018, the dominant transmission chain of E11 was D5 genotype, which was consistent with the genotyping results of enterovirus infection cases in the same period (31). Simultaneously, an outbreak of neonatal E11 infection occurred in Shunde city, adjacent to Guangzhou city, in 2019 (4). Notably, we have reported that E11 sequences in clinical cases from 2017 to 2019 are closely related to E11 sequences continuously sampled from local sewage from 2018 to 2019 (31), which reflects that clinical infection is most likely caused by hidden circulation in the population, and E11 may spread and circulate among different cities. This indicates that our long-term sewage surveillance provides an early warning for possible large-scale outbreaks of E11 in Guangdong Province of China. However, due to the long-term prevalence or silent transmission of E11 in the environment and population in the past decade, this may lead to the improvement of the level of herd immunity. Therefore, it may lead to the decline of the prevalence of E11 in the population and environment, which is reflected in the decrease of the number of E11 detected in 2020 and 2021. Genotype E E11 was found in the domestic sewage isolates in 2021, but no case of infection with this genotype was reported in Guangzhou. It is necessary to continue to monitor E11 in cases and sewage (31).

E6 is also one of the most frequently detected EV serotypes in sewage. The environmental surveillance of sewage in Shandong province has proved that E6 has been the main serotype for some years (32, 33). Our study also showed that E6 was the most common serotype in sewage in 2013 and 2015. However, since the outbreak of COVID-19 in December 2019, many countries have actively taken preventive and control measures to limit the spread of severe acute respiratory syndrome coronavirus 2 (the virus that causes COVID-19), such as wearing masks, washing hands frequently, and maintaining social distance, which has greatly reduced crowd gathering and communication, thereby, reducing the incidence of many other infectious diseases (34, 35). Therefore, the detection rate of most EVs in sewage, including E6 and E11, decreased significantly in 2020 and 2021.

CVB3 and CVB5 exhibit a trend of alternating popularity. CVB5 was detected in large numbers in 2013 to 2014 and 2017 to 2018, while CVB3 was most frequently detected in 2015 to 2016 and 2020 to 2021, and CVB3 was the serotype with the highest detection rate during 2020 to 2021, which may influence the level of herd immunity in the population. Among CVBs, CVB5 is the most common enterovirus serotype, which may cause human infection (36–38). Phylogenetic analysis showed that the global CVB5 could be divided into five genotypes of A to E. In China, most CVB5 strains belong to genotype E. For example, the CVB5 strains in sewage from 2013 to 2021 in our study mainly belong to the genotype E, but it is relatively scattered abroad (38). In China, CVB5 infection has led to more and more reports of severe hand, foot, and mouth disease, sporadic central nervous system disease, and aseptic meningitis (36, 39). Genotype C is usually associated with sporadic cases and outbreaks, including cases in Shandong Province in 2005 and 2009 (36, 40). In 2009 and 2012, CVB5 also caused aseptic meningitis outbreaks in Henan, Yunnan, Guangdong, and Changchun (38). Another study on sewage surveillance in Zhoukou City, Henan Province in 2018 showed that the nucleotide identity of CVB5 from AFP case surveillance and environmental surveillance was 94.7% to 99.6%, indicating that CVB5 from AFP case surveillance and environmental surveillance belonged to the same genotype and highly correlated (41). However, China has not included CVB5 infection in the disease surveillance system, so it is difficult to assess the disease burden comprehensively. Therefore, environmental surveillance provides important and useful information for case surveillance (41).

The results of environmental surveillance in Shandong Province and Guangdong Province of China showed that CVB3 was a serotype with high detection rate (14, 15). The wide variety of transmission routes and high proportion of silent infection make the prevention and control of CVB3 infection more difficult, which is one of the reasons for the prevalence of CVB3 in Guangdong Province. CVB3 of genotype E is widely distributed around the world, including Asia, Europe, America, and Oceania, indicating that it may have strong transmission capacity. In the past, CVB3 of genotype D was the main prevalence in China, while CVB3 of other genotypes was only prevalent in some countries and regions (42). Interestingly, in our study, CVB3 isolated before 2019 belonged to genotype E closely related to the isolates from Shandong Province. However, since 2020, from the perspective of evolutionary relationship, genotype C has been closely related to French isolates. Thus, we conclude that it may be an imported genotype. At present, our research on CVB3 is insufficient, and environmental surveillance still needs to be strengthened.

In conclusion, outbreaks of diseases caused by EVs with severe consequences occur frequently worldwide, and both neonates and the elderly are more susceptible to EVs than other age groups. In the absence of a comprehensive and systematic EV disease surveillance system, environmental surveillance is a powerful and effective tool to investigate the silent circulation of EVs in the population. It is difficult to master the prevalence of EVs in the community caused by asymptomatic infections through disease surveillance, which reflects the significance of long-term environmental surveillance.

## MATERIALS AND METHODS

### Sewage sample collection.

We obtained totally 432 raw sewage samples from January 2013 to December 2021 from the primary sedimentation tank at the Liede wastewater treatment plant located in the northern part of Guangzhou City, China. Four samples (1 L per sample) were collected from the inlets of the primary sedimentation tanks on a routine basis each month. Samples were collected, transported, and refrigerated in the laboratory within 2 h for sample processing.

### Virus concentration.

We used negatively charged membrane filters (mixed cellulose ester membrane filters; Advantec Co. Ltd., Tokyo, Japan) and sonication methods to concentrate viruses in sewage samples (43). The collected sewage samples were centrifuged at 4°C and 3,000 rpm for 30 min to precipitate impurities in the water samples. Subsequently, MgCl_2_ (0.05 M) was added to the supernatant, and the pH was adjusted to 3.5 to 4.0 with HCl. The filter papers and the negatively charged membranes were installed on the filter in turn, and then the positive pressure pump was connected to slowly filter the sewage sample, so that the virus was adsorbed on the negatively charged membrane. To elute the viruses, the virus-adsorbed filters were cut into pieces and sonicated for 1 min in 10 mL of 3% beef extract solution (pH 9.6) (Meilunbio, Dalian, China). The filter was eluted twice to obtain the first and second eluents, and then the pH was adjusted to 7.0 with HCl. Following centrifugation at 4°C and 3,000 rpm for 30 min, the supernatant was aspirated and passed through a 0.22 μm syringe filter (Merck Millipore, USA) to remove bacteria and fungi.

### Virus isolation.

Human rhabdomyosarcoma and laryngeal epidermoid carcinoma cells used for the virus isolation in our experiments were obtained from National Polio laboratory at the Chinese Center for Disease Control and Prevention. The cells were cultured in 24-well plates and six parallel wells were set, of which 200 μL of the first and 200 μL of second eluents were pipetted into four and two wells, respectively. A total of 200 μL of each concentrated eluate was used to inoculate standard monolayers of cells and cultured in a 37°C, 5% CO_2_ incubator. We recorded wells with cytopathic lesions by microscopy for 7 consecutive days. Independent molecular typing of positive culture supernatants was conducted (44).

### RNA extraction and molecular typing.

Viral RNA was extracted from the collected cultures using a QIAamp Viral RNA minikit (Qiagen, Valencia, CA, USA), and nested reverse transcription-PCR (RT-PCR) was performed according to a previously developed method (45). Briefly, cDNA was synthesized by using a Qiagen OneStep RT-PCR kit (final volume, 20 μL) with 1 μL each of cDNA primers (AN32, AN33, AN34, and AN35). Following incubation at 37°C for 60 min, 2.5 μL cDNA was then used in the first PCR (PCR1: final volume, 25 μL) with a Hot Start PCR Master Mix kit (Invitrogen, USA) with 1 μL (each) of primers 224 and 222, targeting a highly conserved motif in the viral protein (*VP*)*3* and *VP1* regions, respectively.

The first-round PCR (PCR1) was carried out under the following conditions: 95°C for 30 s, 42°C for 30 s, and 60°C for 45 s. After 40 cycles of amplification, an additional elongation step at 60°C for 6 min was done, and then 2.5 μL of PCR1 products was used as a template in the second-round PCR with 1 μL (each) of primers AN88 and AN89, targeting a partial *VP1* region. After 40 cycles of amplification (95°C for 30 s, 40°C for 20 s, and 72°C for 15 s) and an additional elongation step at 60°C for 6 min, the PCR products were analyzed on 1.5% agarose gels, and the positive products (approximately 350 bp long) were purified using a QIAquick PCR purification kit (Qiagen) and sent for sequencing using either primer AN88 or AN89. The sequences were analyzed with the Basic Local Alignment Search Tool server at the National Center for Biotechnology Information, and the serotype of each isolate was determined according to a previously described molecular typing method (46).

### Sequence data sets and phylogenetic analysis.

Partial *VP1* sequences of enterovirus strains from the study were compared with sequences of representative strains available in GenBank (Table 2). BioEdit 7.0.9.1 software was used for nucleotide and amino acid identity analysis (36). These sequences were analyzed by the neighbor-joining method implemented in the MEGA-X program. The credibility of phylogenetic relationships was evaluated through 1,000 bootstrap replications (47).

**TABLE 2:** Representative sequences of CVB3 and CVB5 from around the world

Serotype	Genotype	Name	GenBank no.	Yr	Place
CVB3	A	CB3/Sweden/1987	M16572	1987	Sweden
	B	GA/America/1956	AY673831	1956	America
	C	0450-76/GER/1976	DQ093624	1976	Germany
	C	CF1930611-05/France/2005	AM236938	2005	France
	C	CF1851041-05/France/2005	AM236933	2005	France
	D	94196/SD/CHN/1994	GQ329766	1994	Shandong, China
	D	20011693/TW/CHN/2001	JQ390190	2001	Taiwan, China
	D	20031159/TW/CHN/2003	JQ390191	2003	Taiwan, China
	E	08201/SD/CHN/2008	GQ329765	2008	Shandong, China
	E	H019Y/SD/CHN/2008	GQ329756	2008	Shandong, China
	E	37010408199/SD/CHN/2008	GQ329767	2008	Shandong, China
CVB5	A	Faulkner/Sweden	AF114383	—	Sweden
	A	6051/Sweden/1974	GU300056	1974	Ecuador
	A	Inferred ancestor/Sweden	GU323577	—	Sweden
	B	98388/SD/CHN/1998	GQ329771	1998	Shandong, China
	B	CF1247P_FRA_99/France/1999	HF948117	1999	France
	C	4458.91/Australia/1991	FJ868335	1991	Australia
	C	STU29_DEU_09/Germany/2009	HF948277	2009	Germany
	C	X67706.1 UK/1992	X67706	1992	United Kingdom
	D	4804/Kyrgyzstan/1992	GU300059	1992	Kyrgyzstan
	D	1603/Finland/82/1997	AJ004643	1997	Finland
	E	CF2380481-05/France/2005	AM236951	2005	France
	E	JB141230179/Guangdong/2012	KC867094	2012	Guangdong, China

### Data availability.

The complete *VP1* sequences of 55 CVB3 strains and 111 CVB5 strains described in this study have been deposited in the GenBank database under accession numbers OQ351960 to OQ352014 and OQ352015 to OQ352125.

## References

[1] Oberste MS, Maher K, Kilpatrick DR, Pallansch MA. 1999. Molecular evolution of the human enteroviruses: correlation of serotype with VP1 sequence and application to picornavirus classification. J Virol 73:1941–1948. doi:10.1128/JVI.73.3.1941-1948.1999. 9971773 PMC104435

[2] Oberste MS, Maher K, Kilpatrick DR, Flemister MR, Brown BA, Pallansch MA. 1999. Typing of human enteroviruses by partial sequencing of VP1. J Clin Microbiol 37:1288–1293. doi:10.1128/JCM.37.5.1288-1293.1999. 10203472 PMC84754

[3] Lefkowitz EJ, Dempsey DM, Hendrickson RC, Orton RJ, Siddell SG, Smith DB. 2018. Virus taxonomy: the database of the International Committee on Taxonomy of Viruses (ICTV). Nucleic Acids Res 46:D708–D717. doi:10.1093/nar/gkx932. 29040670 PMC5753373

[4] Lu J, Kang M, Zeng H, Zhong Y, Fang L, Zheng X, Liu L, Yi L, Lin H, Peng J, Li C, Zhang Y, Sun L, Luo S, Xiao J, Munnink BBO, Koopmans MPG, Wu J, Zhang Y, Zhang Y, Song T, Li H, Zheng H. 2020. Tracking echovirus eleven outbreaks in Guangdong, China: a metatranscriptomic, phylogenetic, and epidemiological study. Virus Evol 6:veaa029. doi:10.1093/ve/veaa029. 32411392 PMC7211399

[5] Bodilsen J, Mens H, Midgley S, Brandt CT, Petersen PT, Larsen L, Hansen BR, Luttichau HR, Helweg-Larsen J, Wiese L, Ostergaard C, Storgaard M, Nielsen H, Danish Study Group of Infections of the Brain. 2021. Enterovirus meningitis in adults: a prospective nationwide population-based cohort study. Neurology 97:e454–e463. doi:10.1212/WNL.0000000000012294. 34088872

[6] Messacar K, Schreiner TL, Maloney JA, Wallace A, Ludke J, Oberste MS, Nix WA, Robinson CC, Glode MP, Abzug MJ, Dominguez SR. 2015. A cluster of acute flaccid paralysis and cranial nerve dysfunction temporally associated with an outbreak of enterovirus D68 in children in Colorado, USA. Lancet 385:1662–1671. doi:10.1016/S0140-6736(14)62457-0. 25638662

[7] Mulders MN, Serhan F, Goodson JL, Icenogle J, Johnson BW, Rota PA. 2017. Expansion of surveillance for vaccine-preventable diseases: building on the global polio laboratory network and the global measles and rubella laboratory network platforms. J Infect Dis 216:S324–S330. doi:10.1093/infdis/jix077. 28838191 PMC5853980

[8] Diop OM, Kew OM, de Gourville EM, Pallansch MA. 2017. The global polio laboratory network as a platform for the viral vaccine-preventable and emerging diseases laboratory networks. J Infect Dis 216:S299–S307. doi:10.1093/infdis/jix092. 28838192 PMC5853949

[9] Wilkinson AL, Diop OM, Jorba J, Gardner T, Snider CJ, Ahmed J. 2022. Surveillance to track progress toward polio eradication - worldwide, 2020–2021. MMWR Morb Mortal Wkly Rep 71:538–544. doi:10.15585/mmwr.mm7115a2. 35421079 PMC9020859

[10] Zhang Y, Yan D, Zhu S, Wen N, Li L, Wang H, Liu J, Ye X, Ding Z, Wang D, Zhu H, Chen L, Hou X, An H, Liang X, Luo H, Kew O, Xu W. 2010. Type 2 vaccine-derived poliovirus from patients with acute flaccid paralysis in China: current immunization strategy effectively prevented its sustained transmission. J Infect Dis 202:1780–1788. doi:10.1086/657410. 21050127

[11] Chiu ML, Luo ST, Chen YY, Chung WY, Duong V, Dussart P, Chan YF, Perera D, Ooi MH, Thao NTT, Truong HK, Lee MS. 2020. Establishment of Asia-Pacific Network for Enterovirus Surveillance. Vaccine 38:1–9. doi:10.1016/j.vaccine.2019.09.111. 31679864

[12] Harvala H, Benschop KSM, Berginc N, Midgley S, Wolthers K, Simmonds P, Feeney S, Bailly JL, Mirand A, Fischer TK, on behalf of the ENPEN Hospital-Based Surveillance N. 2021. European Non-Polio Enterovirus Network: introduction of hospital-based surveillance network to understand the true disease burden of non-polio enterovirus and parechovirus infections in Europe. Microorganisms 9:1827. doi:10.3390/microorganisms9091827. 34576722 PMC8469463

[13] Duintjer Tebbens RJ, Diop OM, Pallansch MA, Oberste MS, Thompson KM. 2019. Characterising the costs of the Global Polio Laboratory Network: a survey-based analysis. BMJ Open 9:e023290. doi:10.1136/bmjopen-2018-023290. PMC6347914 30670511

[14] Wang H, Tao Z, Li Y, Lin X, Yoshida H, Song L, Zhang Y, Wang S, Cui N, Xu W, Song Y, Xu A. 2014. Environmental surveillance of human enteroviruses in Shandong Province, China, 2008–2012: serotypes, temporal fluctuation and molecular epidemiology. Appl Environ Microbiol 80:4683–4691. doi:10.1128/AEM.00851-14. 24837389 PMC4148804

[15] Zheng H, Lu J, Zhang Y, Yoshida H, Guo X, Liu L, Li H, Zeng H, Fang L, Mo Y, Yi L, Chosa T, Xu W, Ke C. 2013. Prevalence of nonpolio enteroviruses in the sewage of Guangzhou city, China, from 2009 to 2012. Appl Environ Microbiol 79:7679–7683. doi:10.1128/AEM.02058-13. 24096418 PMC3837794

[16] Hamisu AW, Blake IM, Sume G, Braka F, Jimoh A, Dahiru H, Bonos M, Dankoli R, Mamuda Bello A, Yusuf KM, Lawal NM, Ahmed F, Aliyu Z, John D, Nwachukwu TE, Ayeni MF, Gumede-Moeletsi N, Veltsos P, Giri S, Praharaj I, Metilda A, Bandyopadhyay A, Diop OM, Grassly NC. 2020. Characterizing environmental surveillance sites in Nigeria and their sensitivity to detect poliovirus and other enteroviruses. J Infect Dis 225:1377–1386. doi:10.1093/infdis/jiaa175. PMC9016446 32415775

[17] Perepliotchikov Y, Ziv-Baran T, Hindiyeh M, Manor Y, Sofer D, Moran-Gilad J, Stephens L, Mendelson E, Weil M, Bassal R, Anis E, Singer SR, Kaliner E, Cooper G, Majumdar M, Markovich M, Ram D, Grotto I, Gamzu R, Martin J, Shulman LM. 2021. Inferring numbers of wild poliovirus excretors using quantitative environmental surveillance. Vaccines (Basel) 9. 10.3390/vaccines9080870 PMC8402366 34451995

[18] Ozawa H, Yoshida H, Usuku S. 2019. Environmental surveillance can dynamically track ecological changes in enteroviruses. Appl Environ Microbiol 85:e01604-19. doi:10.1128/AEM.01604-19. 31585989 PMC6881801

[19] Brinkman NE, Fout GS, Keely SP. 2017. Retrospective surveillance of wastewater to examine seasonal dynamics of enterovirus infections. mSphere 2:e00099-17. doi:10.1128/mSphere.00099-17. 28630939 PMC5471348

[20] Bubba L, Broberg EK, Jasir A, Simmonds P, Harvala H, Redlberger-Fritz M, Nikolaeva-Glomb L, Havlíčková M, Rainetova P, Fischer TK, Midgley SE, Epštein J, Blomqvist S, Böttcher S, Keeren K, Bujaki E, Farkas Á, Baldvinsdóttir GE, Morley U, De Gascun C, Pellegrinelli L, Piralla A, Martinuka O, Zamjatina N, Griškevičius A, Nguyen T, Dudman SG, Numanovic S, Wieczorek M, Guiomar R, Costa I, Cristina T, Bopegamage S, Pastuchova K, Berginc N, Cabrerizo M, González-Sanz R, Zakikhany K, Hauzenberger E, Benschop K, Duizer E, Dunning J, Celma C, McKenna J, Feeney S, Templeton K, Moore C, Cottrell S, Enterovirus Study Collaborators. 2020. Circulation of non-polio enteroviruses in 24 EU and EEA countries between 2015 and 2017: a retrospective surveillance study. Lancet Infect Dis 20:350–361. doi:10.1016/S1473-3099(19)30566-3. 31870905

[21] Abedi GR, Watson JT, Nix WA, Oberste MS, Gerber SI. 2018. Enterovirus and parechovirus surveillance - United States, 2014–2016. MMWR Morb Mortal Wkly Rep 67:515–518. doi:10.15585/mmwr.mm6718a2. 29746455 PMC5944979

[22] Harvala H, Jasir A, Penttinen P, Pastore Celentano L, Greco D, Broberg E. 2017. Surveillance and laboratory detection for non-polio enteroviruses in the European Union/European Economic Area, 2016. Euro Surveill 22:16–807. doi:10.2807/1560-7917.ES.2017.22.45.16-00807. PMC5718392 29162204

[23] Pham NTK, Thongprachum A, Trinh QD, Okitsu S, Komine-Aizawa S, Shimizu H, Hayakawa S, Ushijima H. 2018. Detection and genetic characterization of enterovirus strains circulating among children with acute gastroenteritis in Japan during 2014–2016. Infect Genet Evol 61:16–19. doi:10.1016/j.meegid.2018.03.009. 29540319

[24] Xing W, Liao Q, Viboud C, Zhang J, Sun J, Wu JT, Chang Z, Liu F, Fang VJ, Zheng Y, Cowling BJ, Varma JK, Farrar JJ, Leung GM, Yu H. 2014. Hand, foot, and mouth disease in China, 2008–12: an epidemiological study. Lancet Infect Dis 14:308–318. doi:10.1016/S1473-3099(13)70342-6. 24485991 PMC4035015

[25] Ji T, Han T, Tan X, Zhu S, Yan D, Yang Q, Song Y, Cui A, Zhang Y, Mao N, Xu S, Zhu Z, Niu D, Zhang Y, Xu W. 2019. Surveillance, epidemiology, and pathogen spectrum of hand, foot, and mouth disease in mainland of China from 2008 to 2017. Biosafety and Health 1:32–40. doi:10.1016/j.bsheal.2019.02.005.

[26] Tang J, Yoshida H, Ding Z, Tao Z, Zhang J, Tian B, Zhao Z, Zhang L. 2014. Molecular epidemiology and recombination of human enteroviruses from AFP surveillance in Yunnan, China from 2006 to 2010. Sci Rep 4:6058. doi:10.1038/srep06058. 25317568 PMC5377527

[27] Majumdar M, Sharif S, Klapsa D, Wilton T, Alam MM, Fernandez-Garcia MD, Rehman L, Mujtaba G, McAllister G, Harvala H, Templeton K, Mee ET, Asghar H, Ndiaye K, Minor PD, Martin J. 2018. Environmental surveillance reveals complex enterovirus circulation patterns in human populations. Open Forum Infect Dis 5:ofy250. doi:10.1093/ofid/ofy250. 30377626 PMC6201154

[28] Lizasoain A, Burlandy FM, Victoria M, Tort LFL, da Silva EE, Colina R. 2018. An environmental surveillance in Uruguay reveals the presence of highly divergent types of human enterovirus species C and a high frequency of species A and B types. Food Environ Virol 10:343–352. doi:10.1007/s12560-018-9351-7. 29907902

[29] Li J, Yan D, Chen L, Zhang Y, Song Y, Zhu S, Ji T, Zhou W, Gan F, Wang X, Hong M, Guan L, Shi Y, Wu G, Xu W. 2019. Multiple genotypes of Echovirus 11 circulated in mainland China between 1994 and 2017. Sci Rep 9:10583. doi:10.1038/s41598-019-46870-w. 31332200 PMC6646367

[30] Su T, Zhou Y, Zhu Y, Liu Z, Yang F, Yang S, Yu Z, Guo C, Ma S. 2015. Molecular characterization of a new human echovirus 11 isolate associated with severe hand, foot and mouth disease in Yunnan, China, in 2010. Arch Virol 160:2343–2347. doi:10.1007/s00705-015-2496-x. 26100404

[31] Huang S, Zhang W, Chen M, Guo X, Zhu S, Li C, Zeng H, Fang L, Zhang Y, Zheng H, Deng X. 2022. Temporal fluctuation and genetic characterization of echovirus-11 isolates upon surveillance of domestic sewage in Guangzhou City, China. Chinese J Virol 38:1108–1116.

[32] Tao Z, Song Y, Wang H, Zhang Y, Yoshida H, Ji S, Xu A, Song L, Liu Y, Cui N, Ji F, Li Y, Chen P, Xu W. 2012. Intercity spread of echovirus 6 in Shandong Province, China: application of environmental surveillance in tracing circulating enteroviruses. Appl Environ Microbiol 78:6946–6953. doi:10.1128/AEM.01861-12. 22843520 PMC3457494

[33] Tao Z, Wang H, Li Y, Xu A, Zhang Y, Song L, Yoshida H, Xu Q, Yang J, Zhang Y, Liu Y, Feng L, Xu W. 2011. Cocirculation of two transmission lineages of echovirus 6 in Jinan, China, as revealed by environmental surveillance and sequence analysis. Appl Environ Microbiol 77:3786–3792. doi:10.1128/AEM.03044-10. 21478313 PMC3127621

[34] Luciani L, Ninove L, Zandotti C, Nougairede A. 2021. COVID-19 pandemic and its consequences disrupt epidemiology of enterovirus meningitis, South-East France. J Med Virol 93:1929–1931. doi:10.1002/jmv.26785. 33482046 PMC8014593

[35] Zomahoun DJ, Burman AL, Snider CJ, Chauvin C, Gardner T, Lickness JS, Ahmed JA, Diop O, Gerber S, Anand A. 2021. Impact of COVID-19 pandemic on global poliovirus surveillance. MMWR Morb Mortal Wkly Rep 69:1648–1652. doi:10.15585/mmwr.mm695152a4. 33382673 PMC9191906

[36] Chen P, Tao Z, Song Y, Liu G, Wang H, Liu Y, Song L, Li Y, Lin X, Cui N, Xu A. 2013. A coxsackievirus B5-associated aseptic meningitis outbreak in Shandong Province, China in 2009. J Med Virol 85:483–489. doi:10.1002/jmv.23478. 23212939

[37] Rezig D, Yahia AB, Abdallah HB, Bahri O, Triki H. 2004. Molecular characterization of coxsackievirus B5 isolates. J Med Virol 72:268–274. doi:10.1002/jmv.10579. 14695669

[38] Rao Q, Long S, He W, Jiang H, Sun Q, Zhang Z. 2021. Genome characteristics of the VP1 region and prediction of the secondary structure of the proteins of coxsackievirus B5. Chinese J Virol 37:106–114.

[39] Hu Y, Yang F, Du J, Zhang T, Xue Y, Jin Q. 2012. Coxsackievirus B5, associated with neurological hand, foot and mouth disease, China. J Infect 65:189–191. doi:10.1016/j.jinf.2012.03.021. 22484273

[40] Hu Y, Zhao R, Xue Y, Yang F, Jin Q. 2012. Full genome sequence of a novel coxsackievirus B5 strain isolated from neurological hand, foot, and mouth disease patients in China. J Virol 86:11408–11409. doi:10.1128/JVI.01709-12. 22997425 PMC3457159

[41] Zhang M, Yang J, Lu H, Xiao J, Zhang Y, Zhang L, Lu M, Zhang Y. 2021. Analyses of environmental-monitoring results of enteroviruses in Henan Province, China in 2018. Chinese J Virol 37:1112–1118.

[42] Yang Q, Zeng H, Zheng H, Yan D, Zhu S, Song Y, Wang D, Han Z, Li J, Zhang Y, Xu W. 2022. Novel analyses of the genetic characteristics and identification of genotype E coxsackievirus B3 strains in Guangdong Province, China, in 2020. Chinese J Virol 38:57–63.

[43] Iwai M, Yoshida H, Matsuura K, Fujimoto T, Shimizu H, Takizawa T, Nagai Y. 2006. Molecular epidemiology of echoviruses 11 and 13, based on an environmental surveillance conducted in Toyama Prefecture, 2002–2003. Appl Environ Microbiol 72:6381–6387. doi:10.1128/AEM.02621-05. 16957267 PMC1563678

[44] Xu W, Zhang Y. 2016. Isolation and characterization of vaccine-derived polioviruses, relevance for the global polio eradication initiative. Methods Mol Biol 1387:213–226. doi:10.1007/978-1-4939-3292-4_10. 26983736

[45] Nix WA, Oberste MS, Pallansch MA. 2006. Sensitive, seminested PCR amplification of VP1 sequences for direct identification of all enterovirus serotypes from original clinical specimens. J Clin Microbiol 44:2698–2704. doi:10.1128/JCM.00542-06. 16891480 PMC1594621

[46] Kroneman A, Vennema H, Deforche K, V D Avoort H, Penaranda S, Oberste MS, Vinje J, Koopmans M. 2011. An automated genotyping tool for enteroviruses and noroviruses. J Clin Virol 51:121–125. doi:10.1016/j.jcv.2011.03.006. 21514213

[47] Bisseux M, Colombet J, Mirand A, Roque A, Abravanel F, Izopet J, Archimbaud C, Peigue H, Debroas D, Bailly J, Henquell C. 2018. Monitoring human enteric viruses in wastewater and relevance to infections encountered in the clinical setting: a one-year experiment in central France, 2014 to 2015. Euro Surveill 23:17–237. doi:10.2807/1560-7917.ES.2018.23.7.17-00237. PMC5824128 29471623

